# New insights into bioaugmented removal of sulfamethoxazole in sediment microcosms: degradation efficiency, ecological risk and microbial mechanisms

**DOI:** 10.1186/s40168-023-01741-5

**Published:** 2024-02-29

**Authors:** Jianfei Chen, Xiuli Chen, Ying Zhu, Shuang Yan, Shuguang Xie

**Affiliations:** 1 Fujian Key Laboratory of Pollution Control & Resource Reuse, College of Environmental and Resource Sciences, Fujian Normal University, Fuzhou, 350007 China; 2 State Key Joint Laboratory of Environmental Simulation and Pollution Control, College of Environmental Sciences and Engineering, Peking University, Beijing, 100871 China

**Keywords:** Bioaugmentation, Sulfonamide, Antibiotic resistance genes (ARGs), DNA-stable isotope probing, Metagenomics

## Abstract

**Background:**

Bioaugmentation has the potential to enhance the ability of ecological technology to treat sulfonamide-containing wastewater, but the low viability of the exogenous degraders limits their practical application. Understanding the mechanism is important to enhance and optimize performance of the bioaugmentation, which requires a multifaceted analysis of the microbial communities. Here, DNA-stable isotope probing (DNA-SIP) and metagenomic analysis were conducted to decipher the bioaugmentation mechanisms in stabilization pond sediment microcosms inoculated with sulfamethoxazole (SMX)-degrading bacteria (*Pseudomonas* sp. M2 or *Paenarthrobacter* sp. R1).

**Results:**

The bioaugmentation with both strains M2 and R1, especially strain R1, significantly improved the biodegradation rate of SMX, and its biodegradation capacity was sustainable within a certain cycle (subjected to three repeated SMX additions). The removal strategy using exogenous degrading bacteria also significantly abated the accumulation and transmission risk of antibiotic resistance genes (ARGs). Strain M2 inoculation significantly lowered bacterial diversity and altered the sediment bacterial community, while strain R1 inoculation had a slight effect on the bacterial community and was closely associated with indigenous microorganisms. *Paenarthrobacter* was identified as the primary SMX-assimilating bacteria in both bioaugmentation systems based on DNA-SIP analysis. Combining genomic information with pure culture evidence, strain R1 enhanced SMX removal by directly participating in SMX degradation, while strain M2 did it by both participating in SMX degradation and stimulating SMX-degrading activity of indigenous microorganisms (*Paenarthrobacter*) in the community.

**Conclusions:**

Our findings demonstrate that bioaugmentation using SMX-degrading bacteria was a feasible strategy for SMX clean-up in terms of the degradation efficiency of SMX, the risk of ARG transmission, as well as the impact on the bacterial community, and the advantage of bioaugmentation with *Paenarthrobacter* sp. R1 was also highlighted﻿.

Video Abstract

**Supplementary Information:**

The online version contains supplementary material available at 10.1186/s40168-023-01741-5.

## Background

Sulfonamides (SA) are widely used in the prevention and treatment of human and animal diseases because of their broad-spectrum bacteriostatic property [1]. A considerable proportion of the produced SA can be discharged into the receiving environment via pharmaceutical wastewater, livestock and aquaculture wastewaters, and domestic sewage [1–3], which results in the wide distribution of SA in surface water, sediment, soil, groundwater, and even in drinking water [4–6]. SA are of great eco-environmental concern due to their acute toxicity to aquatic organisms and their persistence in the environment and bioaccumulation in the biological chain, thus were considered as highly toxic drugs in “Environmentally Classified Pharmaceuticals 2009” [7]. Sulfamethoxazole (SMX) is usually used as a model SA compound due to its ubiquity in nature and medium-high ecological risk [4, 8]. The natural attenuation of SA usually proceeds slowly and incompletely in contaminated environments, so it is of great significance to develop strategies to achieve the rapid and complete clean-up of SA [9, 10].

Microbial degradation is the major pathway in the process of SA clean-up in the environments; thus, bioaugmentation-based remediation is an effective approach to accelerate the attenuation of SA in situ [11]. Bioaugmentation has been successfully implemented in many fields such as soil remediation and activated sludge treatment, and it effectively enhances the removal efficiency of pollutants such as pesticides, polychlorinated biphenyl, and polycyclic aromatic hydrocarbons (PAHs) [12–15]. Plenty of microorganisms capable of degrading SA have been isolated from diverse environments, and their potential applications in the clean-up of SA-contaminated environments have been preliminarily evaluated [16–18]. Bioaugmentation with exogenous SA-degrading bacteria may promote the removal of SMX, but their low viability in the bioaugmented systems limits the full-scale application [18, 19]. Understanding the dynamics of exogenous SA-degrading bacteria in the bioaugmentation process, as well as their interactions with indigenous microorganisms, and revealing the active microbes involved in the in-situ degradation of SA, are helpful to solve the dilemma of low viability of exogenous functional bacteria in the bioaugmentation systems [20]; however, relevant studies are still lacking. Additionally, exploring the change of microbial community as well as the enrichment and transmission of antibiotic resistance genes (ARGs) associated with bioaugmentation is also of great importance during the bioaugmentation application [17, 21].

In our previous work, two efficient SA-degraders, *Pseudomonas* sp. M2 (Proteobacteria) and *Paenarthrobacter* sp. R1 (Actinobacteria), were isolated from fishpond sediments [22]. *Pseudomonas* sp. M2 and *Paenarthrobacter* sp. R1 can rapidly degrade three typical SA compounds (SMX, sulfadiazine, and sulfamethazine) in pure cultures, and they were speculated as the potential candidates for the clean-up of SA-contaminated environments [22], awaiting experimental validation. *Pseudomonas* sp. M2 and *Paenarthrobacter* sp. R1 are not only phylogenetically distinct, but also have different catalytic mechanisms for SA biotransformation. *Paenarthrobacter* sp. R1 catalyzes the *ipso*-hydroxylation of SA by the monooxygenase encoded by the *sadA* gene, whereas *Pseudomonas* sp. M2 degrades SA independent of *sadA* gene (not present on M2 genome) [22]. In addition, only SA resistance gene, *sul1* gene, was detected in *Paenarthrobacter* sp. R1, while *Pseudomonas* sp. M2 genome contained various ARGs [22]. SA-degrading bacteria identified or isolated for now mainly belong to phyla Proteobacteria and Actinobacteria [23–28]. Hence, revealing the bioaugmentation mechanisms of the two SA-degraders with different phylogenetic characteristics and SA degradation mechanisms will help develop a bioaugmentation approach for SA-contaminated environments and optimize the selection of exogenous microorganisms.

In the present study, stabilization pond sediment microcosms were established to evaluate the potential of *Pseudomonas* sp. M2 or *Paenarthrobacter* sp. R1 to clean up SMX. Moreover, an approach integrating molecular ecological network analyses, metagenomics, and DNA-stable isotope probing (DNA-SIP) was employed to explore the microbial mechanisms of the bioaugmentation process. The main goals of the present study were to (1) evaluate the bioaugmentation efficacy in terms of sulfonamide removal efficiency and antibiotic resistance genes transmission risk and (2) reveal the bioaugmentation mechanisms based on bacterial community temporal dynamics, interaction networks, active degraders, and assimilation pathway.

## Materials and methods

### Sediment collection

The sediments for the bioaugmentation microcosm study were collected from an ecological stabilization pond (treating wastewater from a livestock and poultry breeding farm) in Ya’an City, Sichuan Province in November 2020. Detailed information on the stabilization pond sediments was described in our previous studies, including physicochemical properties, SA concentrations, and antibiotic removal capacity [29, 30]. After transferring to the laboratory, the sediments were immediately stored at 4 °C before carrying out SA degradation and DNA-SIP experiments.

### Cultivation of SA-degrading bacteria

*Pseudomonas* sp. M2 and *Paenarthrobacter* sp. R1 were previously isolated from fishpond sediments in Xiamen City, Fujian Province, and they had high SA-degrading ability [22]. These two strains might be potential candidates for the clean-up of SA-contaminated environments because they were able to rapidly dissipate typical SA in pure cultures [22]. *Pseudomonas* sp. M2 and *Paenarthrobacter* sp. R1 were stored in glycerol solution (30% v/v) at −80 °C and recultivated according to a previous study [31]. Briefly, the preserved strains were successively cultured in R_2_A solid medium, minimal salt liquid medium [22], and R_2_A liquid medium (all supplemented with 50 mg/L SMX), and then, the bacterial suspensions (OD_600_ = 2) were obtained by culture solution centrifugation (5000 rpm, 5 min), cleaning, and re-suspension (using sterile normal saline).

### Bioaugmentation microcosms

**Fig. 1:**
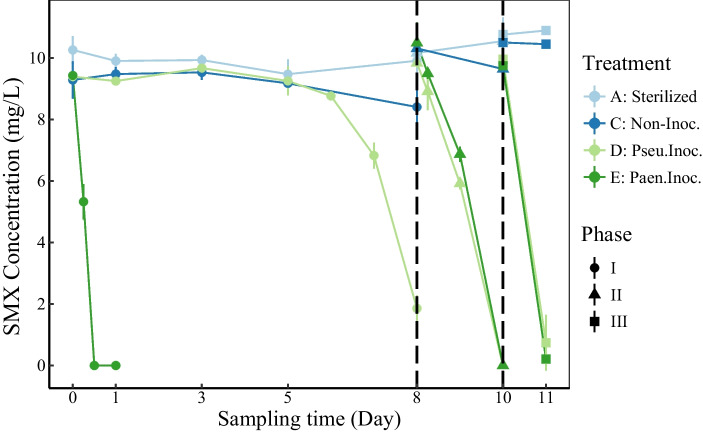
Degradation characteristics of SMX in sediment microcosms. Data are means ± standard deviation, *n* = 3. Treatments A, C, D, and E represent sterilized control, non-inoculated control, inoculated with *Pseudomonas* sp. M2, and inoculated with *Paenarthrobacter* sp. R1, respectively. Phases I, II, and III represent the three different SMX addition periods, namely days 0–8, days 8–10, and days 10–11, respectively

Microcosms were established with the antibiotics-contaminated sediment and simulated synthetic wastewater under aerobic conditions. Each microcosm was prepared as follows: 2 g of sediment (dry weight, < 0.9 mm) was added to 20 mL simulated synthetic wastewater (Additional file 1: Table S1), modified by referring to the in situ physicochemical properties. Sodium acetate and ammonium chloride were added to simulate the total organic carbon and total nitrogen concentrations of in situ water samples). A total of 5 treatments were conducted, including (A) sterilized control (designated as sterilized), (B) non-SMX-amended control (designated as non-SMX), (C) non-inoculated control (designated as non-Inoc.), (D) inoculated with *Pseudomonas* sp. M2 (designated as Pseu.Inoc.), and (E) inoculated with *Paenarthrobacter* sp. R1 (designated as Paen.Inoc.). The microcosms with treatments A, B, C, D, and E were set up with 3, 14, 14, 21, and 21 replicates, respectively. All sediment microcosms were incubated at 25 °C and 160 rpm in the dark for 11 days, and 10 mg/L SMX (10 mg SMX powder was pre-added to 1 L simulated synthetic wastewater, and dissolution was promoted by ultrasonication) was added to the microcosms with treatments A, C, D, and E at days 0, 8, and 10 (days 0–8, days 8–10, and days 10–11 were defined as phases I, II, and III, respectively). Cell suspensions of strains M2 and R1 were pre-added into the synthetic wastewater (with final OD_600_ = 0.01) for the microcosms with treatments D and E, respectively. During the incubation, liquid samples were collected for the quantification of SMX (for each treatment, 100 μL was collected from each of the three microcosms, the sampling time was as shown in Fig. 1). The sacrificial sampling method was used to obtain sediment samples (two microcosms from treatments B and C, and three microcosms from treatments D and E) at days 0, 1, 3, 5, 8, 10, and 11 for molecular analyses, and samples were stored at −80 ℃ before analysis.

### Sulfamethoxazole quantification

The concentration of SMX in each liquid sample was analyzed by high-performance liquid chromatography (HPLC, Agilent u3000) equipped with a Venusil XBP C18 column (Agela Technologies) as described previously [30]. Before HPLC test, the liquid sample was mixed with 90% methanol, and then passed through a 0.22-μm filter. The mobile phase consisted of acetonitrile and 0.025% of formic acid in water (v/v = 7/3, at 0.8 mL/min), and the detection wavelength was 268 nm [32].

### Molecular analyses

#### DNA extraction and real-time qPCR assay of related genes

DNA was extracted from sediment samples using the PowerSoil DNA kit (Qiagen) following the manufacturer’s protocol. Real-time qPCR reactions were conducted to assess the number of bacterial 16S rRNA gene, SA resistance genes (*sul1* and *sul2* genes), and the degradation monooxygenase encoding gene (*sadA* gene) according to our previous study [32]. The primer sets and conditions for qPCR were summarized in Supplementary Information (Additional file 1: Table S2 and Additional file 2: Supplementary Methods).

#### 16S rRNA gene Illumina MiSeq sequencing and raw data processing

The bacterial 16S rRNA gene V4-V5 region was amplified using primer set 515F and 907R with a unique barcode for each sample and then was subjected to high-throughput sequencing on an Illumina MiSeq platform (300 paired-end, Shanghai Majorbio Bio-pharm Technology Co., Ltd.) according to the manufacturer’s protocols [33]. The processing of the raw reads followed the QIIME2 pipeline including diversity analysis and taxonomic classification (version 2020.11) [34]. The detailed information is summarized in Additional file 2: Supplementary Methods.

#### Shotgun metagenomic sequencing and data analysis

DNA samples from each treatment (except sterilized controls) on day 11 were collected (three replicate samples were mixed) for metagenomic shotgun sequencing (150 paired-end) on an Illumina NovaSeq 6000 platform (Shanghai Majorbio Bio-pharm Technology). The functions, especially ARGs and mobile genetic elements (MGEs), of metagenomics data were annotated based on de novo pipeline as described in our previous study [35]. Binned genomes were obtained and annotated following the MetaWRAP pipeline [36]. The detailed methods are described in Additional file 2: Supplementary Methods.

### DNA-SIP experiments

DNA-SIP was performed to identify the active microorganisms involved in SMX degradation in the microcosms inoculated with *Pseudomonas* sp. M2 or *Paenarthrobacter* sp. R1. The microcosms were set up and incubated as abovementioned, and ^13^C-labeled and unlabeled DNAs (when SMX removal efficiency reached about 80%) were obtained by proliferating the target microbiome with SMX-(phenyl-^13^C_6_) and unlabeled SMX as carbon sources (10 mg/L), respectively. DNA samples were precisely divided into 12 fractions with diverse buoyancy densities (BD) based on CsCl density gradient ultracentrifugation according to the DNA-SIP protocol and our recent study (detailed information is summarized in Additional file 1: Table S5 and Additional file 2) [30, 37]. After retrieval, the number of bacterial 16S rRNA gene copies in each fraction was quantified by qPCR using a primer set of 515F and 907R. DNAs of 3–10 fractions (based on density fractionation characteristic of bacterial 16S rRNA gene) from both ^13^C-labeled and unlabeled samples were selected for 16S rRNA gene Illumina Miseq sequencing. DNA extraction, qPCR assay, Illumina Miseq sequencing, and data analysis were performed as above-described.

### Bioinformatics and statistical analysis

Statistical analysis (*p* < 0.05) and visualization were performed in the R software (Version 4.2.3), unless otherwise noted. The method of data-pre-processing before statistical analysis is described in Additional file 2: Supplementary Methods. A one-way analysis of variance (ANOVA) with the least significant difference (LSD) test was carried out to examine the significance of the difference in the microbial alpha diversity and related genes number among treatments (vegan and agricolae R package, bonferroni *p* value correction). Beta-diversity was assessed with principal coordinate analysis (PCoA) and PERMANOVA based on weighted UniFrac distance metric. ALDEx2 (centered log-ratio transformed, Wilcoxon test, *P* values were corrected by Benjamini-Hochberg) was used to identify microbial groups with significant difference in abundance among treatments in the bioaugmentation experiment [38, 39]. The co-occurrence ecological network was constructed following the online MENA pipeline based on Pearson’s correlation analysis (http://ieg2.ou.edu/MENA) [40], and Gephi (Version 0.9.7) was applied to visualize the network [41]. Zi-Pi (within-module and among-module connectivity) plot as well as network structural robustness calculation based on natural connectivity was applied to identify and verify keystone populations that had great influences on network stability [40, 42, 43] (detailed methods are described in Additional file 2: Supplementary Methods).

## Results

### Characteristics of sulfamethoxazole degradation

The removal efficiencies were lower than 3.5% in the sterilized control microcosms after 8 days’ incubation, so abiotic processes contributed slightly to SMX elimination. The non-inoculated sediment microcosm showed a certain SMX degradation capacity, but the removal efficiency was only about 10% in 8 days (Fig. 1). The two studied strains (*Pseudomonas* sp. M2 and *Paenarthrobacter* sp. R1) both accelerated SMX biodegradation in sediment microcosms, especially strain *Paenarthrobacter* sp. R1. In the microcosms inoculated with *Paenarthrobacter* sp. R1, SMX was completely removed within half a day, and a high rate of SMX degradation was even maintained when SMX was re-added at days 8 and 10 (with removal efficiencies of nearly 100% within two days). In the microcosms inoculated with *Pseudomonas* sp. M2, the SMX removal efficiency increased by 70% compared to the non-inoculated microcosms on day 8, and the degradation efficiencies at days 10 and 11 were similar to those in the microcosms with *Paenarthrobacter* sp. R1 when SMX was re-added. Therefore, the bioaugmentation using both *Pseudomonas* sp. M2 and *Paenarthrobacter* sp. R1 could significantly improve the SMX biodegradation efficiency in SMX-amended sediment microcosms.

### Temporal dynamics and interaction networks of bacterial communities

**Fig. 2:**
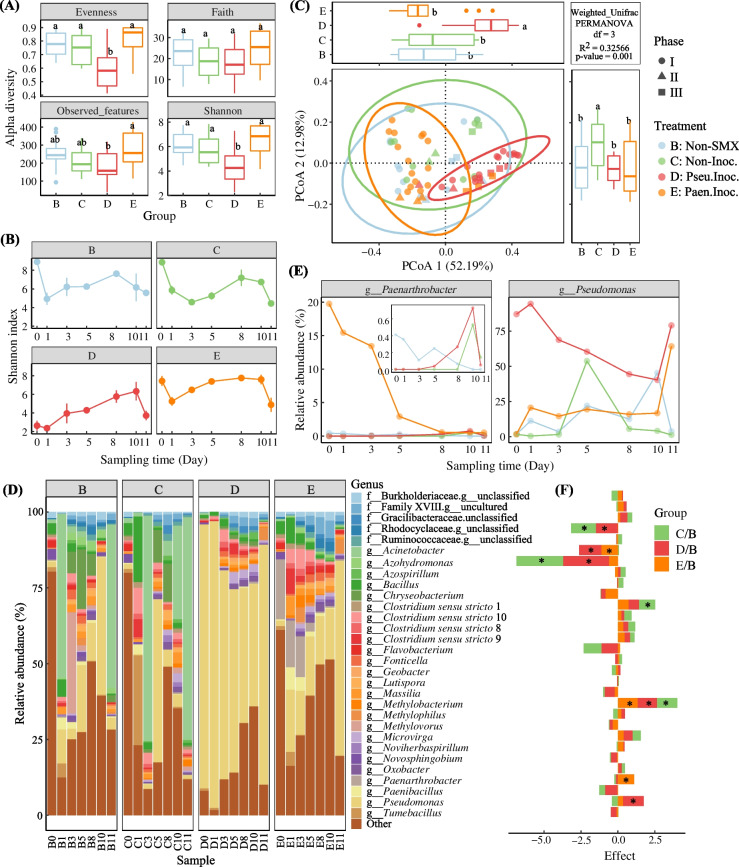
The boxplots show the bacterial alpha diversity indices in sediments (**A**). Shannon index during incubation (**B**). The PCoA plot based on the Weighted_Unifrac distance (**C**). Relative abundance of the 30 largest bacterial genera (**D**). Temporal dynamics of *Pseudomonas* sp. M2 and *Paenarthrobacter* sp. R1 (**E**). Comparison of the different genus distribution between SMX-amended treatments (treatments C, D, and E) and non-SMX treatment (treatment B) (**F**). All taxonomic groups except for the top 30 were merged into the “Others” group. ANOVA with an LSD test (*P* < 0.05) indicates statistically significant differences denoted by different letters for each assessed parameter (**A**, **C**). ALDEx2 was used to identify the microbial groups with significant difference between treatments, and “*” indicates *P* < 0.05 (centered log-ratio transformed, Wilcoxon test, *P* values were corrected by Benjamini-Hochberg) (**F**)

Inoculation with *Pseudomonas* sp. M2 significantly decreased evenness, observed features and Shannon index (alpha diversity) in sediment, while inoculation with *Paenarthrobacter* sp. R1 significantly increased observed features of the bacterial communities (Fig. 2A, ANOVA, *P* < 0.05). The Shannon index of each treatment firstly decreased (on day 1) and then increased with incubation time (Fig. 2B). Bacterial community compositions were significantly different among treatments based on Weighted_Unifrac similarity (PERMANOVA, *R*^*2*^ = 0.33, *P* = 0.001) (Fig. 2C). The samples from treatments Pseu.Inoc. and Non-Inoc. were distinctly separated from others on the first and second axes, respectively, while the samples from treatments Paen.Inoc. and Non-SMX showed similar bacterial community structures.

The addition of SMX or the inoculation of R1 and M2 showed no significant effect on the phylum composition (Additional file 2: Fig. S1), but the profile of bacterial composition at genus level indicated different distributions of the dominant microbial members (Fig. 2D, F). *Methylobacterium* was more abundant in all of the three SMX-added treatments, and *Clostridium *sensu stricto 1, *Paenarthrobacter*, and *Pseudomonas* were respectively more abundant in treatments Non-Inoc., Pseu.Inoc., and Paen.Inoc., compared to non-SMX treatment (ALDEx2, *P* < 0.05). On the contrary, the relative abundance of f_Rhodocyclaceae.g_unclassified (an unclassified member affiliated within Rhodocyclaceae), *Acinetobacter*, or *Azohydromonas* was significantly decreased in the SMX-added microcosms (*P* < 0.05). Besides, compared to non-inoculated control, *Paenarthrobacter*, *Flavobacterium*, *Azohydromonas*, and f_Rhodocyclaceae.g_unclassified were enriched in treatment Paen.Inoc. (*P* < 0.05), and *Pseudomonas* was enriched in treatment Pseu.Inoc. (*P* < 0.05), while the abundance of *Acinetobacter* decreased in both inoculated treatments (Additional file 2: Fig. S2). In addition, the relative abundance of *Paenarthrobacter* in the non-inoculated sediment microcosm samples was less than 0.2%, but it reached about 20% after inoculation and then decreased with the incubation time (Fig. 2E). The decrease in *Paenarthrobacter* abundance in treatment Paen.Inoc. might be related to the absence of SMX for a long time, and the abundance increased slightly after the re-addition of SMX on day 8. Interestingly, the relative abundance of *Paenarthrobacter* in treatments non-Inoc. and Pseu.Inoc. also increased after 10 days of incubation, and the growth induction of *Paenarthrobacter* in treatment Pseu.Inoc. was stronger. *Pseudomonas* dominated in all treatments, and its abundance decreased with the degradation of SMX in *Pseudomonas* sp. M2-inoculated microcosms, but increased in both inoculated microcosms after the re-addition of SMX.

**Fig. 3:**
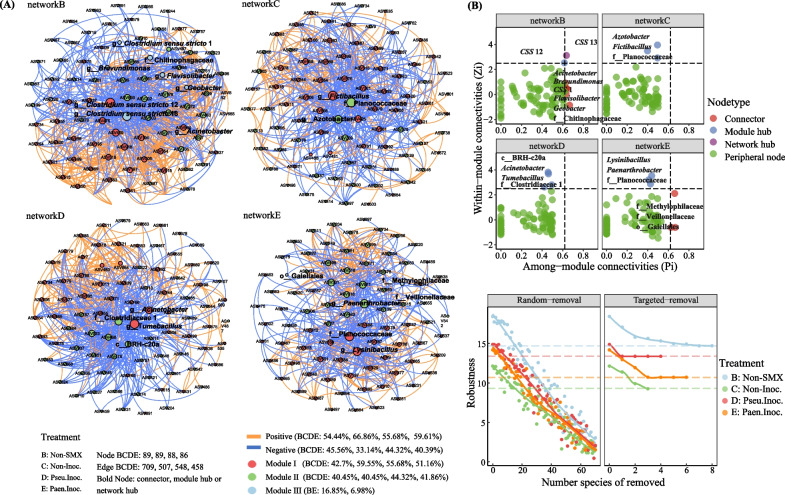
Co-occurrence network analysis showing the biological interactions in each treatment based on pairwise Pearson’s correlations between ASVs (*ρ* > 0.6) (**A**). The color and size of each node represent module class and degree value, respectively. Zi-Pi plot showing the distribution of bacterial ASVs based on their topological roles (**B**). Zi and Pi are within-module connectivity and among-module connectivity. Network hubs: nodes with Zi > 2.5 and Pi > 0.62; Module hubs: nodes with Zi > 2.5 and Pi ≤ 0.62; Connectors: nodes with Zi ≤ 2.5 and Pi > 0.62; Peripheral nodes: nodes with Zi ≤ 2.5 and Pi ≤ 0.62. *CSS*: *Clostridium *sensu stricto. Structural robustness (estimated by natural connectivity) of random removal and targeted removal (the impacts of the loss of the keystone species on robustness of treatments B, C, D, and E were respectively consistent with the loss of 19, 19, 12, and 19 species, reaching 20%, 23%, 10%, and 25%) (**C**)

The interactions among sediment microorganisms were explored using the correlation-based co-occurrence ecological networks (Fig. 3). The addition of SMX reduced the complexity (average degree decreased), closeness (average clustering coefficient decreased and average path distance increased), and modularity (module number decreased) of the networks, but promoted the cooperative relationship (the proportion of positive correlation increased), which was mitigated by the inoculation of *Pseudomonas* sp. M2 but exacerbated by the inoculation of *Paenarthrobacter* sp. R1 (Fig. 3A and Additional file 1: Table S3). In addition, 8, 3, 4, and 6 ASVs were determined as the keystone taxa (having a great influence on network stability) in treatments non-SMX, non-Inoc., Pseu.Inoc., and Paen.Inoc., respectively, according to the scatter plot of within-module connectivity (Zi) and among-module connectivity (Pi) (Fig. 3B and Additional file 1: Table S4). The effects of random loss of nodes in the network and targeted loss of the keystones on the structural robustness also showed the importance of the proposed keystones (especially module hubs) on the microbial stability, where the impacts of the loss of the keystone species on robustness of treatments non-SMX, non-Inoc., Pseu.Inoc., and Paen.Inoc. were respectively consistent with the loss of 19, 19, 12, and 19 species, reaching 20%, 23%, 10%, and 25% (Fig. 3C). No common keystone taxa appeared in the four treatments except *Acinetobacter* and f_Planococcaceae.g_unclassified (an unclassified member affiliated within Planococcaceae), which were simultaneously identified as keystone taxa in treatments non-SMX and Pseu.Inoc., and treatments non-Inoc. and Paen.Inoc., respectively. *Paenarthrobacter* was a module hub of treatment Paen.Inoc. (with the degree of 39), and it was positively correlated with ASV458 (*Tumebacillus*), ASV488 (*Clostridium sensu stricto* 12), and ASV489 (*Clostridium sensu stricto* 13), but negatively correlated with other 36 ASVs. However, *Pseudomonas* was not identified as a keystone taxon in treatment Pseu.Inoc., and only showed negative and positive correlations with ASV569 (o__DTU014.g__unclassified) and ASV971 (*Acinetobacter*), respectively.

### Abundance of functional genes, antibiotic resistance genes, and mobile genetic elements

**Fig. 4:**
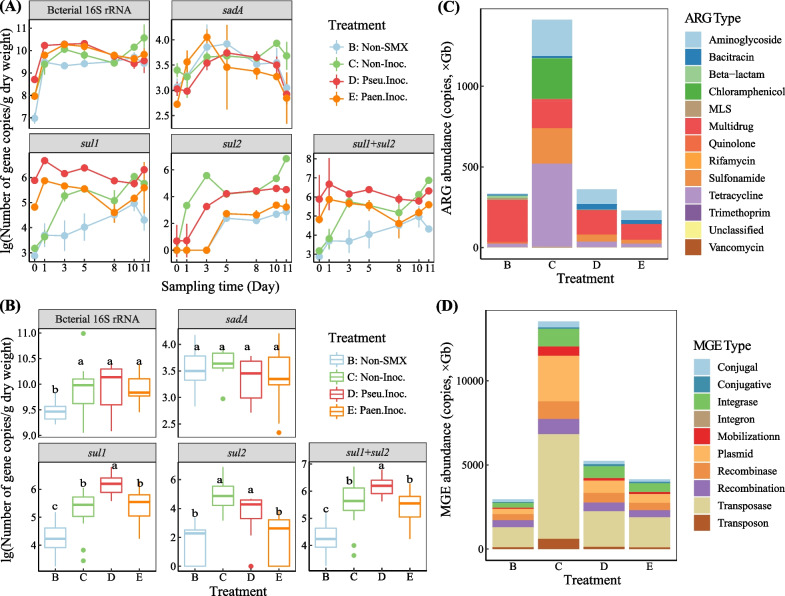
Temporal dynamics (**A**) and inter-treatment differences (**B**) of bacterial 16S rRNA gene, sulfonamide degradation monooxygenase encoding gene (*sadA* gene) and resistance genes (*sul1* and *sul2* genes) based on qPCR results. The relative abundance of antibiotic resistance genes (ARGs) (**C**) and mobile genetic elements (MGEs) (**D**) in sediments based on shot gun metagenomic sequencing. The discrete points outside the box in **B** with values apparently above or below the data range represent “outliers” for the data

The number of bacterial 16S rRNA gene rapidly increased after one day of incubation and then remained relatively stable (Fig. 4A). Besides, the amendment of SMX significantly increased the number of bacteria (Fig. 4B). The number of SA biodegradation monooxygenase encoding gene (*sadA* gene) firstly increased but then decreased with incubation time, and there was no significant difference in *sadA* gene copies among the four treatments (Fig. 4A, B). The number of *sul1* gene (SA resistance gene) increased slowly and rapidly in treatments non-SMX and non-Inoc. during the incubation period, respectively. Moreover, the number of *sul1* gene in treatments Pseu.Inoc. and Paen.Inoc. mounted up immediately after the inoculation of *Pseudomonas* sp. M2 or *Paenarthrobacter* sp. R1, followed by a decrease with the degradation of SMX, and slightly increased after the re-addition of SMX. In the end of the incubation period, the rank of *sul1* gene number in the four treatments was treatment Pseu.Inoc. > non-Inoc. > Paen.Inoc. > non-SMX. The number of another SA resistance gene, *sul2* gene, in all these treatments increased with incubation time as well, and *sul2* gene copies in treatments non-Inoc. and Pseu.Inoc. were significantly higher than those in treatments non-SMX and Paen.Inoc. (*p* < 0.05). Additionally, after 11 days of incubation, the rank of the total number of *sul1* and *sul2* genes in the four treatments was treatment non-Inoc. > Pseu.Inoc. > Paen.Inoc. > non-SMX, suggesting that the inoculation of SMX-degraders (especially *Paenarthrobacter* sp. R1) could abate the enrichment of SA resistance genes induced by SMX pollution.

Metagenomic analysis further demonstrated that bioaugmentation using SMX-degraders could abate ARG accumulation and transmission risk (represented by the abundance of ARGs and MGEs) (Fig. 4C and D). The abundance (coverage, ×Gb) of both ARGs and MGEs in treatment non-Inoc. was higher than that in the other three treatments, as 4.2 and 5.3, 3.9 and 2.1, and 6.2 and 2.8 times as that in treatments non-SMX, Pseu.Inoc., and Paen.Inoc., respectively. Moreover, it was noteworthy that the addition of SMX but without exogenous SMX-degrader not only resulted in the accumulation of SA resistance genes, but also promoted the abundance of tetracycline, multidrug, chloramphenicol, and aminoglycoside resistance genes, as well as the abundance of MGE such as integrase, plasmid and transposase.

### Active sulfamethoxazole degraders revealed by DNA-SIP

**Fig. 5:**
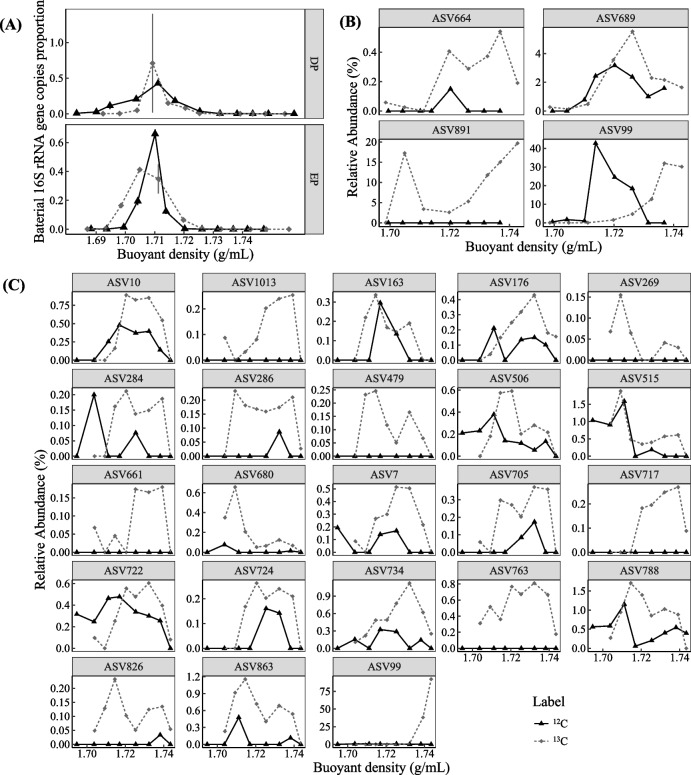
Relative abundance of bacterial 16S rRNA gene (**A**) and potential SMX-degraders in the treatments inoculated with *Paenarthrobacter* sp. R1 (**B**) and *Pseudomonas* sp. M2 (**C**) along buoyant density gradients

To further reveal the microbial mechanism of promoted SMX removal by exogenous degrading bacteria, DNA-SIP was conducted to identify active microorganisms involved in SMX biodegradation during the bioaugmentation process. A total of 23 and 4 bacterial types, dominating in the heavy DNA fractions of the ^13^C-SMX labeled treatments but not in the corresponding fractions of unlabeled control, were identified as the functional SMX degraders in *Pseudomonas* sp. M2 and *Paenarthrobacter* sp. R1 inoculated microcosms, respectively (Fig. 5, Additional file 1: Table S6, Additional file 2: Figs. S3 and S4). The diversity of SMX-degrading bacteria was relatively high in *Pseudomonas* sp. M2 inoculated microcosm, but the average abundance of most microbes was less than 1%, and *Paenarthrobacter* was the dominant SMX-degrader (Fig. 5C, ASV99, with relative abundance of 92% in fraction 10 from ^13^C-labeled treatment). Although *Pseudomonas* was highly abundant in sediment after inoculation, it was not labeled by ^13^C-SMX (Fig. S3, ASV978), indicating that *Pseudomonas* sp. M2 might only improve SMX removal by promoting the metabolism of SMX by indigenous microorganisms. *Paenarthrobacter*, *Methylophilus*, *Methylobacterium*, and *Caulobacter* (ASV99, ASV891, ASV689, and ASV664) were identified as the active SMX-degraders in *Paenarthrobacter* sp. R1 inoculated microcosm, and *Paenarthrobacter* was the most abundant functional microorganism participating in SMX degradation, as well (Fig. 5B). Therefore, *Paenarthrobacter* sp. R1 promoted the removal of SMX mainly by directly participating in the biodegradation of SMX.

### Prediction of sulfamethoxazole assimilation pathway

**Fig. 6:**
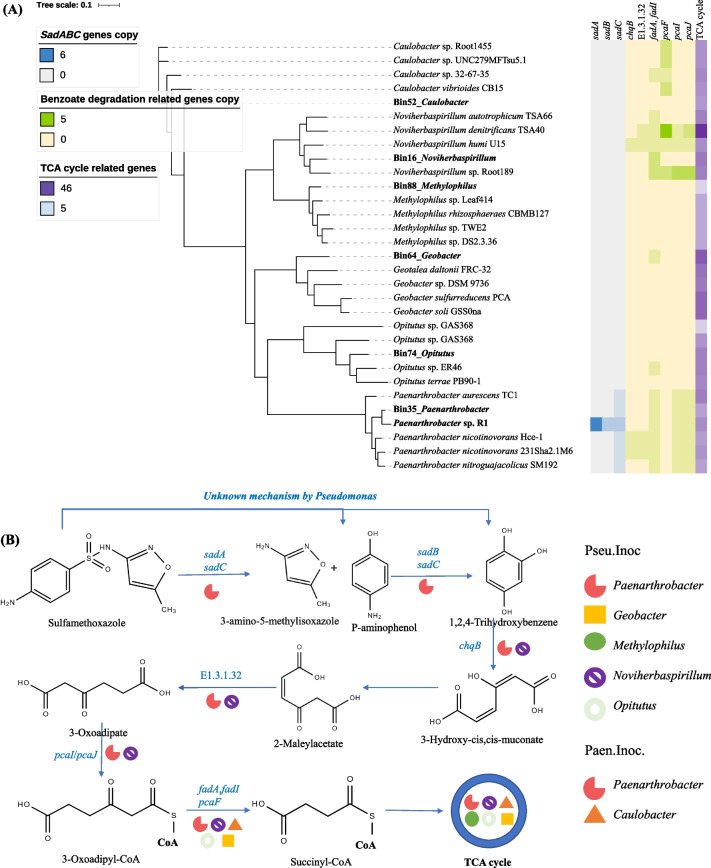
Phylogenetic tree of the putative SA-degraders-related bins and their reference genomes based on 400 marker genes (**A**) and the proposed SMX assimilation pathway (**B**). *Paenarthrobacter* sp. R1 was the strain used in this study. The products were predicted according to our previous studies [22], KEGG (map00627 and map00362) and the composition of functional genes in the SMX-degraders

A total of 91 assembled genomes (with completeness > 70% and contamination < 10%) were recovered, including the six previously proposed SMX-degraders (Bin16_*Noviherbaspirillum*, Bin35_*Paenarthrobacter*, Bin52_*Caulobacter*, Bin64_*Geobacter*, Bin74_*Opitutus*, and Bin88_*Methylophilus*) (Fig. 6A and Additional file 2: Fig. S5). A putative SMX assimilation pathway was proposed according to the evidence provided by the SMX-degrader genomes and our previous studies (Fig. 6B) [22, 30]. SMX was firstly converted to 1,2,4-trihydroxybenzene by the flavin-dependent monooxygenases and FMN reductase encoded by the *sadABC* genes. Further degradation of 1,2,4-trihydroxybenzene shared similar mechanisms of subsequent degradation of benzoate (KEGG map00362). Briefly, 1,2,4-trihydroxybenzene was converted to succinyl-CoA and eventually entered the TCA cycle by hydroxyquinol 1,2-dioxygenase (*chqB* gene), maleylacetate reductase (E1.3.1.32), 3-oxoadipate CoA-transferase (*pcaI* or *pcaJ* gene), acetyl-CoA acyltransferase (*fadA* and *fadI* gene), and 3-oxoadipyl-CoA thiolase (*pcaF* gene). The SMX initial *ipso*-hydroxylation-related functional genes, *sadABC* genes, were only detected on the *Paenarthrobacter* genomes, indicating its important role in the initial transformation of SMX. Besides, all genes associated with the hypothesized SMX assimilation pathway were annotated on the *Paenarthrobacter* genomes, which was consistent with its dominance in ^13^C-SMX labeled microorganisms in both bioaugmentation systems. Pangenomic analysis showed that *sadA* gene existed only in our isolated strain of *Paenarthrobacter* (*Paenarthrobacter* sp. R1) and was not shared by other *Paenarthrobacter* microorganisms (Fig. 6A). The *Noviherbaspirillum* genome, which contained all the relevant genes except the *sadABC* genes, also played an important role in the *Pseudomonas* sp. M2-inoculated system. Additionally, the other ^13^C-labeled microorganisms were mainly involved in the TCA cycle step (*Caulobacter* in *Paenarthrobacter* sp. R1-inoculated system, and *Geobacter*, *Methylophilus*, and *Opitutus* in *Pseudomonas* sp. M2-inoculated system). Last but also important, although *Pseudomonas* was not labeled by ^13^C-SMX and did not contain the *sadABC* genes, it had been demonstrated to catalyze the *ipso*-hydroxylation of SMX and utilize SMX as the sole carbon source [22]. Therefore, in *Pseudomonas* sp. M2-inoculated systems, the initial transformation of SMX might be also driven by *Pseudomonas*, whose intermediate products were subsequently assimilated by other ^13^C-labeled microorganisms such as *Paenarthrobacter* and *Noviherbaspirillum*.

## Discussion

### Bioaugmentation strategies successfully removed sulfamethoxazole

The natural attenuation of SA usually proceeds slowly and incompletely in contaminated ecosystems, leading to the accumulation of SMX in the environments [9, 10], in line with the result observed in non-bioaugmented sediment microcosms in this study. Microbial degradation is the main pathway for the removal of SA [2, 44], and the key factor impeding fast and complete attenuation of SA is likely the absence or low abundance of potent metabolic microorganisms in contaminated ecosystems, which also accounts for the slow degradation of other organic pollutants (e.g., PAHs) [45, 46]. Bioaugmentation, an effective approach to accelerate the natural attenuation of pollutants by introducing exogenous functional microorganisms, has been successfully implemented in several fields including soil remediation and activated sludge treatment processes [14, 15]. Recently, some researchers have also suggested that bioaugmentation technology can improve the removal of SA from biofilm reactor, membrane bioreactor, and activated sludge [17, 18, 47], but the feasibility and mechanisms of bioaugmentation application in the natural environment (e.g., sediment) still need to be explored. In this study, we demonstrated that bioaugmentation using either of two SMX-degraders could achieve the rapid degradation of SMX in the sediment microcosms, and in particular, *Paenarthrobacter* sp. R1 showed a much better performance. Additionally, the results of repeated addition tests indicated that the exogenous degrading bacteria could enhance the biodegradation of SMX for at least 3 cycles. Noteworthily, *Paenarthrobacter* and *Pseudomonas* could also degrade other structurally distinct SA (sulfadiazine, sulfamethazine, etc.) [22, 48, 49], which made the strains R1 and M2 be suitable for bioaugmented treatment of wastewater contaminated with multiple SA compounds.

### Bioaugmentation strategies successfully lowered ARG transmission risk

The accumulation and transmission of ARGs during antibiotic biotreatment processes is a common challenge. On one hand, the spreading of ARGs is one of the important ecological and environmental issues imposed by antibiotic pollution [1, 50]. On the other hand, the known SA resistance genes are usually shared among the SA-degrading bacteria [48, 51]; thus, the inoculation with exogenous SMX-degrading bacteria risks promoting the accumulation and transmission of ARGs. A previous work pointed out that the number of SA resistance genes was affected by antibiotic treatment in a bioaugmented moving bed biofilm reactor, but no comparison was made between the bioaugmentation group and the control group, and this phenomenon was not elucidated in terms of the genome composition and the dynamic changes of resistance genes [17]. The ability of bioaugmentation process to abate ARGs should be further investigated [21]. As expected, *sul1* gene (one of SA resistance genes) was observed in the genome of *Paenarthrobacter* sp. R1 [22], and *Pseudomonas* is a common host for *sul1* gene [52, 53], which accounted for the immediate increase in *sul1* gene in the two inoculation treatments. Additionally, although s*ul1* gene was not annotated in our previously assembled *Pseudomonas* sp. M2 genome (constrained by sequencing and assembly methods), the quantitative assay results provide evidence for the presence of *sul1* gene [22]. Moreover, we found that although inoculation of exogenous SMX-degraders directly introduced *sul1* gene into the sediments, the introduced ARG abundance (number or coverage) was significantly lower than that caused by SMX accumulation, especially the abundance of total ARGs and MGEs (Fig. 4). Our findings suggested bioaugmentation as a promising treatment strategy for SMX pollution in terms of the high removal efficiency of SMX and the low risk of ARG transmission. Moreover, the superiority of *Paenarthrobacter* sp. R1 was also highlighted.

### Different responses of bacterial communities to strains M2 and R1

The findings of this study contributed to a better understanding of the influence of bioaugmentation using exogenous SMX-degrading bacteria on the microbial ecology in the environment. We found that bioaugmentation using *Pseudomonas* sp. M2 significantly lowered the diversity and changed the structure of the indigenous sediment microbial communities, while bioaugmentation using *Paenarthrobacter* sp. R1 did not cause obvious changes in the overall diversity and structure. The remodeling of indigenous bacterial community structure by exogenous microorganisms is a common phenomenon in the process of bioaugmentation [21, 54]. The long-term existence of SMX made *Pseudomonas* maintain a continuously dominant position in the bioaugmented sediment microcosm, so it had a great impact on the structure of the indigenous microbial communities. However, *Paenarthrobacter* could completely clean up SMX in a very short time, and its abundance decreased after SMX removal because of its obligate metabolism for SMX, which imposed a slight impact on the indigenous communities [46, 55]. In addition, *Paenarthrobacter* was identified as a module hub of the network, indicating that it had established robust linkages with various indigenous microorganisms. The successful insertion of *Paenarthrobacter* into the indigenous microbial network was conducive to the stable existence of foreign bacteria in the bacterial community and the sustainability of SMX clean-up [46]. In contrast, the link between *Pseudomonas* and indigenous microorganisms was not strong, which was consistent with the conclusion that the community keystones were often rare species with low abundance [56, 57]. These fundamental ecological findings could be valuable for the selection of microorganisms for bioaugmentation practice.

### Bioaugmentation mechanisms of exogenous degraders

Despite the satisfactory performance of several bioaugmentation applications to treat SA-containing wastewaters by the addition of exogenous degraders [17, 18, 47], no study has confirmed the activities or degrading capacities of the reintroduced strains in SA degradation. DNA-SIP has found successful applications in exploring active PAHs-degraders in bioaugmented soils [13, 45, 58, 59], and this method can also help to unveil the mechanisms of promoting SMX removal by exogenous degraders. The present study for the first time revealed the mechanism of exogenous degrading bacteria in the bioaugmentation treatment of SMX.

#### Bioaugmentation mechanisms of *Paenarthrobacter* sp. R1

Four bacterial genera (*Paenarthrobacter*, *Methylobacterium*, *Caulobacter*, and *Methylophilus*), especially *Paenarthrobacter*, were identified as the main SMX-degrading microorganism in *Paenarthrobacter* sp. R1 bioaugmentation treatments based on DNA-SIP analysis. *Paenarthrobacter* and *Methylobacterium* have been shown to degrade SMX in pure culture experiments, and they might be the important microbes driving the initial transformation of SMX in the bioaugmentation microcosms [22, 60]. *Methylophilus* can promote the biodegradation of pollutants such as PAHs through co-metabolism, which might also explain its mechanism in SMX degradation [61]. *Caulobacter* strains can degrade PAHs and make it have the potential to metabolize intermediate metabolites of SMX benzene ring [62]. The findings of these microbial degradation capabilities and the conservation of *sadABC* genes in a few Microbacteriaceae and Micrococcaceae SA-degraders supported our hypothesis of SMX assimilation pathways with *Paenarthrobacter* as the core player [22, 60, 62, 63]. *Paenarthrobacter* sp. R1 achieved rapid elimination of SMX by directly participating in the biotransformation process of SMX and cooperating with a few indigenous microorganisms.

#### Bioaugmentation mechanisms of *Pseudomonas* sp. M2

A total of 23 microorganisms were labeled by ^13^C-SMX rather than *Pseudomonas* in *Pseudomonas* sp. M2 bioaugmentation system. Results of DNA-SIP, gene annotation, and pure culture all suggested the important role of *Paenarthrobacter* in SMX removal in *Pseudomonas* sp. M2 bioaugmentation system [22, 64]. Some metagenomic studies have speculated the potential of *Geobacter* and *Bradyrhizobium* for SMX degradation [65, 66], while other ^13^C-labeled bacteria were not associated with SMX biodegradation. More diverse ^13^C-labeled microorganisms might be related to cross-feeding caused by prolonged labeling [67], and these labeled microorganisms might be mainly involved in the degradation of SMX intermediates, which was also consistent with the results of gene annotation. DNA-SIP and genomic assembly evidence suggested that *Pseudomonas* sp. M2 could promote SMX removal by stimulating the SMX-degrading activity of indigenous microorganisms (*Paenarthrobacter*) in the bacterial community. Previous reports on PAHs removal using bioaugmentation treatment also suggested that exogenous degrading bacteria enhanced the biodegradation of pollutants mainly by changing the composition and diversity of in situ degrading bacteria as well as co-metabolism with them, rather than directly participating in the metabolism of pollutants [13, 45, 58].The production of surfactants by exogenous bacteria, like *Pseudomonas*, was a possible reason for the activation of indigenous degrading bacteria [45, 68–70]. Besides, *Pseudomonas* has been proven to catalyze the *ipso*-hydroxylation of sulfonamide based on pure culture experiments in our previous study [22]; thus, it could also achieve SMX assimilation by catalyzing the initial biotransformation of SMX and with the cooperation of other microorganisms. On the whole, the mechanism of *Pseudomonas* sp. M2 enhancing SMX removal revolved two aspects: (1) *Pseudomonas* directly participated in the initial transformation of SMX through unknown mechanisms, and its products were subsequently assimilated by the microorganisms ^13^C-labeled microbes. (2) The addition of *Pseudomonas* induced the growth and SMX degradation activity of *Paenarthrobacter*.

Last but not least, we noted that SMX degradation rates increased in phase II and phase III when SMX was re-added. It has been well-documented that no degradation process happens before the preferred carbon sources are depleted or the substrate concentration is below the threshold value, and the insufficient induction of the catabolic genes is often considered as one of the reasons for this phenomenon [23, 71–74]. In the first phase, the low SMX concentration and the addition of sodium acetate resulted in the low activity of SMX degradation genes of *Pseudomonas*, which further affected the SMX removal efficiencies. After the first phase of induction, the re-addition of SMX could induce a relatively high SMX-degrading genes activity and thus could rapidly attenuate SMX. The mechanism of SMX degradation by *Pseudomonas* and the cooperative relationship between *Pseudomonas* and *Paenarthrobacter* still needs to be further explored.

## Conclusion

In this study, we evaluate the bioaugmentation using *Paenarthrobacter* sp. R1 and *Pseudomonas* sp. M2 from both the perspective of SMX removal efficiency and ARG transmission risk and explored the microbial mechanisms of bioaugmentation process in terms of temporal dynamics of the bacterial community, ecological network, the in-situ degraders, and SMX assimilation pathway. Bioaugmentation using both *Paenarthrobacter* sp. R1 and *Pseudomonas* sp. M2, especially R1, could achieve rapid removal of SMX in sediment microcosms and could last for at least 3 cycles according to the repeated SMX addition tests. Although the inoculation with *Paenarthrobacter* sp. R1 and *Pseudomonas* sp. M2 directly introduced *sul1* gene to the sediments, ultimately, it could significantly abate the risk of ARGs transmission through rapidly degradation of SMX. Due to the dominant position of *Pseudomonas* in the bacterial community, M2 significantly lowered the diversity of the bacterial community and altered its structure, and it was not closely linked with the indigenous microorganisms. On the contrary, *Paenarthrobacter* sp. R1 showed a slight effect on the indigenous bacterial community and established robust linkages with various indigenous microorganisms, which was conducive to its colonization in sediments and its SA-metabolic function. The evidences of DNA-SIP and genomic assembly, as well as our previous pure culture experiment, indicating that *Paenarthrobacter* sp. R1 enhanced SMX removal by directly participating in SMX degradation, while *Pseudomonas* sp. M2 did it by both directly participating in SMX degradation and stimulating SMX-degrading activity of indigenous microorganisms (*Paenarthrobacter*) in the community. Overall, this study demonstrates that bioaugmentation with SMX-degraders, especially *Paenarthrobacter* sp. R1, is a feasible strategy to dissipate SMX.

### Supplementary Information

**Additional file 1: Table S1.** The components of simulated synthetic wastewater. **Table S2.** The primers and annealing conditions for qPCR. **Table S3.** Topological properties of the co-occurrence networks. **Table S4.** The keystone taxa in different treatments based on ASVs’ topological roles. **Table S5.** Buoyancy densities and bacterial 16S rRNA gene copies of SIP fractions. **Table S6.** Taxonomy information on SMX-degraders identified by DNA-SIP.

**Additional file 2: Supplementary Methods.** Real-time q-PCR assay. Computational analysis of 16S rRNA gene high-throughput sequencing. Metagenomics analysis. SIP gradient fractionation. Data pre-processing. Network analysis. **Fig. S1.** Relative abundance of the dominant phyla (all taxonomic groups except for the top 7 were merged into the “Others” group) (A). Comparison of the phylum distribution between SMX-amended treatments (treatments C, D and E) and non-SMX-amended control (treatment B) (B) and between inoculation treatments (treatments D and E) and non-inoculation treatment (treatment C) (C). **Fig. S2.** Comparison of the different genus distribution between inoculation treatments (treatments D and E) and non-inoculation treatment (treatment C). **Fig. S3.** Relative abundance of ASVs along buoyant density gradients from the treatment inoculated with *Pseudomonas* sp. M2. **Fig. S4.** Relative abundance of ASVs along buoyant density gradients from the treatment inoculated with *Paenarthrobacter* sp. R1. **Fig. S5.** Phylogenetic tree using single-copy gene of all assembled bins.

## Data Availability

The raw 16S rRNA gene reads and the shotgun metagenomics data were deposited into the NCBI Sequence Read Archive (SRA) database with the accession number PRJNA974918. Genomes of two isolated strains were deposited to NCBI with project IDs of PRJNA974926 and PRJNA974929.
