# Supplementary material for: New insights into bioaugmented removal of sulfamethoxazole in sediment microcosms: degradation efficiency, ecological risk and microbial mechanisms
Source: Microbiome. 2024 Feb 29;12:43. doi: 10.1186/s40168-023-01741-5 (PMC10903153; doi:10.1186/s40168-023-01741-5)
Supplement: Supplementary file 3 — Additional file 2: Supplementary Methods. Real-time q-PCR assay. Computational analysis of 16S rRNA gene high-throughput sequencing. Metagenomics analysis. SIP gradient fractionation. Data pre-processing. Network analysis. Fig. S1. Relative abundance of the dominant phyla (all taxonomic groups except for the top 7 were merged into the “Others” group) (A). Comparison of the phylum distribution between SMX-amended treatments (treatments C, D and E) and non-SMX-amended control (treatment B) (B) and between inoculation treatments (treatments D and E) and non-inoculation treatment (treatment C) (C). Fig. S2. Comparison of the different genus distribution between inoculation treatments (treatments D and E) and non-inoculation treatment (treatment C). Fig. S3. Relative abundance of ASVs along buoyant density gradients from the treatment inoculated with Pseudomonas sp. M2. Fig. S4. Relative abundance of ASVs along buoyant density gradients from the treatment inoculated with Paenarthrobacter sp. R1. Fig. S5. Phylogenetic tree using single-copy gene of all assembled bins. [file 40168_2023_1741_MOESM2_ESM.docx]

**Supplementary information for**

**New insights into bioaugmented removal of sulfamethoxazole in sediment microcosms: Degradation efficiency, ecological risk and microbial mechanisms**

Jianfei Chen^1,2^, Xiuli Chen^2^, Ying Zhu^2^, Shuang Yan^2^, Shuguang Xie^2,^*

^1^Fujian Key Laboratory of Pollution Control & Resource Reuse, College of Environmental and Resource Sciences, Fujian Normal University, Fuzhou 350007, China

^2^State Key Joint Laboratory of Environmental Simulation and Pollution Control, College of Environmental Sciences and Engineering, Peking University, Beijing 100871, China

* Corresponding author.

Email: xiesg@pku.edu.cn (SG. Xie)

**(Total 14 pages,** **Supplementary methods, references and 5 figures)**

**CONTENTS**

**SUPPLEMENTARY METHODS**

Supplementary Methods: Real-time q-PCR assay

Supplementary Methods: Computational analysis of 16S rRNA gene high-throughput sequencing

Supplementary Methods: Metagenomics analysis

Supplementary Methods: SIP gradient fractionation

Supplementary Methods: Data pre-processing

Supplementary Methods: Network analysis

**SUPPLEMENTARY REFERENCES**

**SUPPLEMENTARY FIGURES**

**Fig. S1.** Relative abundance of the dominant phyla (all taxonomic groups except for the top 7 were merged into the “Others” group) **(A).** Comparison of the phylum distribution between SMX-amended treatments (treatments C, D and E) and non-SMX-amended control (treatment B) **(B)** and between inoculation treatments (treatments D and E) and non-inoculation treatment (treatment C) **(C).**

**Fig. S2.** Comparison of the different genus distribution between inoculation treatments (treatments D and E) and non-inoculation treatment (treatment C).

**Fig. S3.** Relative abundance of ASVs along buoyant density gradients from the treatment inoculated with *Pseudomonas* sp. M2.

**Fig. S4.** Relative abundance of ASVs along buoyant density gradients from the treatment inoculated with *Paenarthrobacter* sp. R1.

**Fig. S5**. Phylogenetic tree using single-copy gene of all assembled bins.

**SUPPLEMENTARY METHODS**

Supplementary Methods: Real-time q-PCR assay

TransStart Top Green qPCR SuperMix kit was used for qPCR assay, and the reaction mixture contained 10 μL 2 × SYBR Green PCR master mix, 0.4 μL of each primer, 1 μL template DNA, and 8.2 μL ddH_2_O. The thermo-cycling steps were performed as follows: 95 °C for 5 min; 40 cycles of 95 °C for 30s, annealing for 30s, and 72 °C for 30s. The primer and annealing temperature for each gene was summarized in Additional file 1: Table S2, and the amplification specificity was evaluated with melting curve analysis (65–95°C, 0.5℃ gradient temperature rise). Three technical replicates were set for each sample. Standard curves were set for quantification. Equal volume nuclease-free ddH_2_O was used as blank control. The standard curves (R^2^ > 0.99, with amplification efficiencies of 80%–110%) were built with plasmids containing the target genes.

Supplementary Methods: Computational analysis of 16S rRNA gene high-throughput sequencing

The raw data of bacterial 16S rRNA gene Illumina MiSeq Sequencing were processed according to QIIME2 pipeline (version 2020.11) [1,2]. In brief, DADA2 pipeline [3], *classify-sklearn* naïve Bayes taxonomic classifier (trained by Silva-132 reference database at a threshold with 97% sequence identity) [4,5], and q2-diversity plugin were respectively used to raw data denoising and high-quality amplicon sequence variants (ASVs) generation, taxonomy affiliation assignment of ASVs, and alpha (Shannon, observed features, evenness, and faith) and beta-diversity (weighted Unifrac distance) estimation (under the sequencing depth of 4,623 sequences).

Supplementary Methods: Metagenomics analysis

The raw reads were quality filtered by KneadData (version 0.6.1) with Trimmomatic v.0.39 (SLIDINGWINDOW:4:20 MINLEN:50) and bowtie2 v2.3.5 (Homo_sapiens genomes removal) [6,7]. The clean reads were assembled into contigs using megahit v.1.2.9 with default k-mers [8], and then QUAST v.5.0.2 was carried out to assess the contigs quality [9]. A total of 5,687,757 contigs with N50 value of 506,633 bp were obtained, and contigs longer than 500 bp were used for downstream analysis including open reading frames (ORFs) prediction, non-redundant gene catalogue clustering and quantification by Prokka v.1.14.6 [10], CD-HIT (with 95% sequence identity and 90% coverage) [11], and Salmon v0.14.1 [12], respectively. The translated protein sequences of the predicted ORFs were aligned against SARG v2.2 database and NR-MGEs database using diamond v0.8.22 BLASP with identity ≥ 80% and coverage ≥ 70% to identify ARG-like and MGE-like ORFs, respectively. The SARG v2.2 database containing ARG sequences from CARD, ARDB and NCBI-NR database (version on July 21, 2016) [13]. The NR-MGE database was extracted from non-redundant (NR) protein sequence database (October, 2020) by keywords (plasmid, integrase, integron, transposase, transposon, recombinase, recombination, conjugative, conjugal or mobilization) according to a previous work [14]. The abundance of ARG- or MGEs-like ORFs was calculated by Eq. (1) [15].

$$Abundance(coverage, \times/Gb)= \sum_{i=1}^{n} \frac{{Ni}_{mapped reads}\times{Li}_{\mathrm{read}}/{Li}_{reference sequence}}{G} (1)$$

Where n is the number of the ARG- or MGE-like ORFs belonging to the same category; *Ni_mapped reads_* is the number of the reads mapped to ARG- or MGEs-like ORFs; *Li_read_* is the length of sequence reads (150 bp); *Li_reference sequence_* (bp) is the length of corresponding ARG- or MGE-like ORFs; G is the size of the data set (Gb).

The binning procedure was performed using MaxBin2 and metaBAT2 in MetaWRAP [16], and the completion and contamination of all the bins were computed by CheckM and kept bins with completion of >70% and contamination <5% [17]. Taxonomy annotation was carried out by annotating the recovered bin-genomes to genome taxonomy database using GTDB toolkits (version 1.0.2, database version 89) based on 120 marker genes [18]. Additional genomes or bins associated with the putative SMX degraders were downloaded from the NCBI database for pangenomic analysis. The ORFs were predicted with Prokka v.1.14.6 [10] and then annotated with KEGG database, ARG database (as above mentioned), and *sadABC* genes database. The GhostKOALA annotation servers were used for KEGG orthologues characterization [19]. *SadABC* genes database was manually collated based on NCBI BLAST, and the GenBank accession number of the sequences were as displayed in our precious work [2]. The phylogenetic tree of all the bins was generated with fasttree2 based on single-copy genes [18], while the phylogenetic tree of bins of the putative SMX degraders and their reference genomes was constructed using PhyloPhlAn based on 400 marker genes [20], and visualized in iTOL [21].

Supplementary Methods: SIP gradient fractionation

For each sample, approximately 2 μg of purified DNA was mixed with CsCl at a final buoyant density (BD) of ~1.725 g/mL in a Quick-Seal polyallomer tube (13 × 51 mm, 5.1 mL, Beckman Coulter, Pasadena, CA, USA). Tubes, after heat-sealed, were centrifuged in an ultracentrifuge (Optima L-100XP, Beckman Coulter, USA) equipped with a Verti 65.2 vertical rotor (Beckman Coulter, USA) at 177,000 × g for 40 h at 20℃ in vacuum. The centrifuged gradients were fractionated into 12 fractions (~450 μL/fraction) through injecting nuclease-free water from the top of the tube using a syringe pump at the rate of 0.45 mL/min. The nD_TC of each fraction was measured using an RA2000 refractometry (Reichert, USA) and then converted to buoyant density (BD, BD = 17.0066×nD_TC^2 – 36.8684×nD_TC + 19.9749). DNA was precipitated from CsCl with polyethylene glycol buffer (800 μL 30% PEG) and mussel glycogen (Roche, 1 μL) for 1–2 h at room temperature, washed with 75% cold ethanol, and finally dissolved in 30 μL nuclease-free water. All DNA samples were stored at -80℃ before further analysis.

Supplementary Methods: Data pre-processing

According to the curve of the bacterial 16S rRNA gene quantity and Shannon index over the incubation time, the number and diversity of bacteria increased apparently on day 1. Therefore, the subsequent statistical analysis of ANOVA and DESeq2 were based on the data of excluding samples on day 0. Besides, low-abundance species were also filtered before DESeq2 analysis (at least 9 samples met more than 10 reads).

Supplementary Methods: Network analysis

We analyzed bacterial networks for the four treatments separately (B: non-SMX-amended control; C: non-inoculated controls; D: inoculated with *Pseudomonas* sp. M2; and E: inoculated with *Paenarthrobacter* sp. R1) following the online MENA pipeline (http://ieg4.rccc.ou.edu/mena/) according to a previous work [22]. To simplify the networks for a better visualization, we removed ASVs occurring in less than 30% of all samples and kept the 92 most abundant ASVs for the further analysis. Pearson’s correlation analysis was employed to test the relationship among ASVs, and the threshold was determined using a random matrix theory-based method (*ρ*>0.6). Node-level topological properties were further calculated on MENA platform (Additional file 1: Table S3). Module, degree centrality, betweenness centrality represent sets of highly inter-connected nodes, the number of paths that connect the local node to other nodes, and potential influence of a particular node on the connections of other nodes, respectively [23]. Interactive Gephi 0.9.2 platform was applied to visualize the networks [24], and Zi-Pi (within-module and among-module connectivity) plot was used to identify key populations based on the topological roles of different nodes in networks (Nodes were divided into four categories: Network hubs: nodes with Zi > 2.5 and Pi > 0.62; Module hubs: nodes with Zi > 2.5 and Pi ≤ 0.62; Connectors: nodes with Zi ≤ 2.5 and Pi > 0.62; and Peripheral nodes: nodes with Zi ≤ 2.5 and Pi ≤ 0.62) [22]. The structural robustness (or invulnerability) was estimated by natural connectivity to determine network stability [25,26]. A proportion of nodes were randomly removed to simulate the random loss of ASVs. Additionally, keystone species were individually removed to simulate targeted removal.

**SUPPLEMENTARY REFERENCES**

1. Bolyen E, Rideout JR, Dillon MR, Bokulich NA, Abnet CC, Al-Ghalith GA, et al. Reproducible, interactive, scalable and extensible microbiome data science using QIIME 2. Nat Biotechnol. 2019;37:852–7.

2. Chen J, Yang Y, Ke Y, Chen X, Jiang X, Chen C, et al. Sulfonamide-metabolizing microorganisms and mechanisms in antibiotic-contaminated wetland sediments revealed by stable isotope probing and metagenomics. Environ Int. 2022;165:107332.

3. Callahan BJ, McMurdie PJ, Rosen MJ, Han AW, Johnson AJA, Holmes SP. DADA2: High-resolution sample inference from Illumina amplicon data. Nat Methods. 2016;13:581–3.

4. Bokulich NA, Kaehler BD, Rideout JR, Dillon M, Bolyen E, Knight R, et al. Optimizing taxonomic classification of marker-gene amplicon sequences with QIIME 2’s q2-feature-classifier plugin. Microbiome. 2018;6:90.

5. Quast C, Pruesse E, Yilmaz P, Gerken J, Schweer T, Yarza P, et al. The SILVA ribosomal RNA gene database project: improved data processing and web-based tools. Nucleic Acids Res. 2012;41:D590–6.

6. Bolger AM, Lohse M, Usadel B. Trimmomatic: a flexible trimmer for Illumina sequence data. Bioinformatics. 2014;30:2114–20.

7. Langmead B, Salzberg SL. Fast gapped-read alignment with Bowtie 2. Nat Methods. 2012;9:357–9.

8. Li D, Liu C, Luo R, Sadakane K, Lam T-W. MEGAHIT: an ultra-fast single-node solution for large and complex metagenomics assembly via succinct de Bruijn graph. Bioinformatics. 2015;31:1674–6.

9. Gurevich A, Saveliev V, Vyahhi N, Tesler G. QUAST: quality assessment tool for genome assemblies. Bioinformatics. 2013;29:1072–5.

10. Seemann T. Prokka: rapid prokaryotic genome annotation. Bioinformatics. 2014;30:2068–9.

11. Fu L, Niu B, Zhu Z, Wu S, Li W. CD-HIT: accelerated for clustering the next-generation sequencing data. Bioinformatics. 2012;28:3150–2.

12. Patro R, Duggal G, Love MI, Irizarry RA, Kingsford C. Salmon provides fast and bias-aware quantification of transcript expression. Nat Methods. 2017;14:417–9.

13. Yin X, Jiang X, Chai B, Li L, Yang Y, Cole JR, et al. ARGs-OAP v2.0 with an expanded SARG database and Hidden Markov Models for enhancement characterization and quantification of antibiotic resistance genes in environmental metagenomes. Bioinformatics. 2018;34:2263–70.

14. Zhang H, Chang F, Shi P, Ye L, Zhou Q, Pan Y, et al. Antibiotic resistome alteration by different disinfection strategies in a full-scale drinking water treatment plant deciphered by metagenomic assembly. Environ Sci Technol. 2019;53:2141–50.

15. Ma L, Xia Y, Li B, Yang Y, Li L, Tiedje JM, et al. Metagenomic assembly reveals hosts of antibiotic resistance genes and the shared resistome in pig, chicken, and human feces. Environ Sci Technol. 2016;50:420–7.

16. Uritskiy GV, DiRuggiero J, Taylor J. MetaWRAP—a flexible pipeline for genome-resolved metagenomic data analysis. Microbiome. 2018;6:158.

17. Parks DH, Imelfort M, Skennerton CT, Hugenholtz P, Tyson GW. CheckM: assessing the quality of microbial genomes recovered from isolates, single cells, and metagenomes. Genome Res. 2015;25:1043–55.

18. Parks DH, Chuvochina M, Waite DW, Rinke C, Skarshewski A, Chaumeil P-A, et al. A standardized bacterial taxonomy based on genome phylogeny substantially revises the tree of life. Nat Biotechnol. 2018;36:996–1004.

19. Kanehisa M, Sato Y, Morishima K. BlastKOALA and GhostKOALA: KEGG tools for functional characterization of genome and metagenome sequences. J Mol Biol. 2016;428:726–31.

20. Segata N, Börnigen D, Morgan XC, Huttenhower C. PhyloPhlAn is a new method for improved phylogenetic and taxonomic placement of microbes. Nat Commun. 2013;4:2304.

21. Letunic I, Bork P. Interactive tree of life (iTOL) v4: recent updates and new developments. Nucleic Acids Res. 2019;47:W256–9.

22. Deng Y, Jiang Y-H, Yang Y, He Z, Luo F, Zhou J. Molecular ecological network analyses. BMC Bioinformatics. 2012;13:113.

23. Chen W, Wen D. Archaeal and bacterial communities assembly and co-occurrence networks in subtropical mangrove sediments under spartina alterniflora invasion. Environ Microbiome. 2021;16:10.

24. Heymann S, Grand BL. Visual analysis of complex networks for business intelligence with Gephi. 17th International Conference on Information Visualisation. 2013, pp 307–312. https://doi.org/10.1109/IV.2013.39.

25. Yuan MM, Guo X, Wu L, Zhang Y, Xiao N, Ning D, et al. Climate warming enhances microbial network complexity and stability. Nat Clim Change. 2021;11:343–8.

26. Peng G, Wu J. Optimal network topology for structural robustness based on natural connectivity. Phys Stat Mech Its Appl. 2016;443:212–20.

**SUPPLEMENTARY FIGURES**


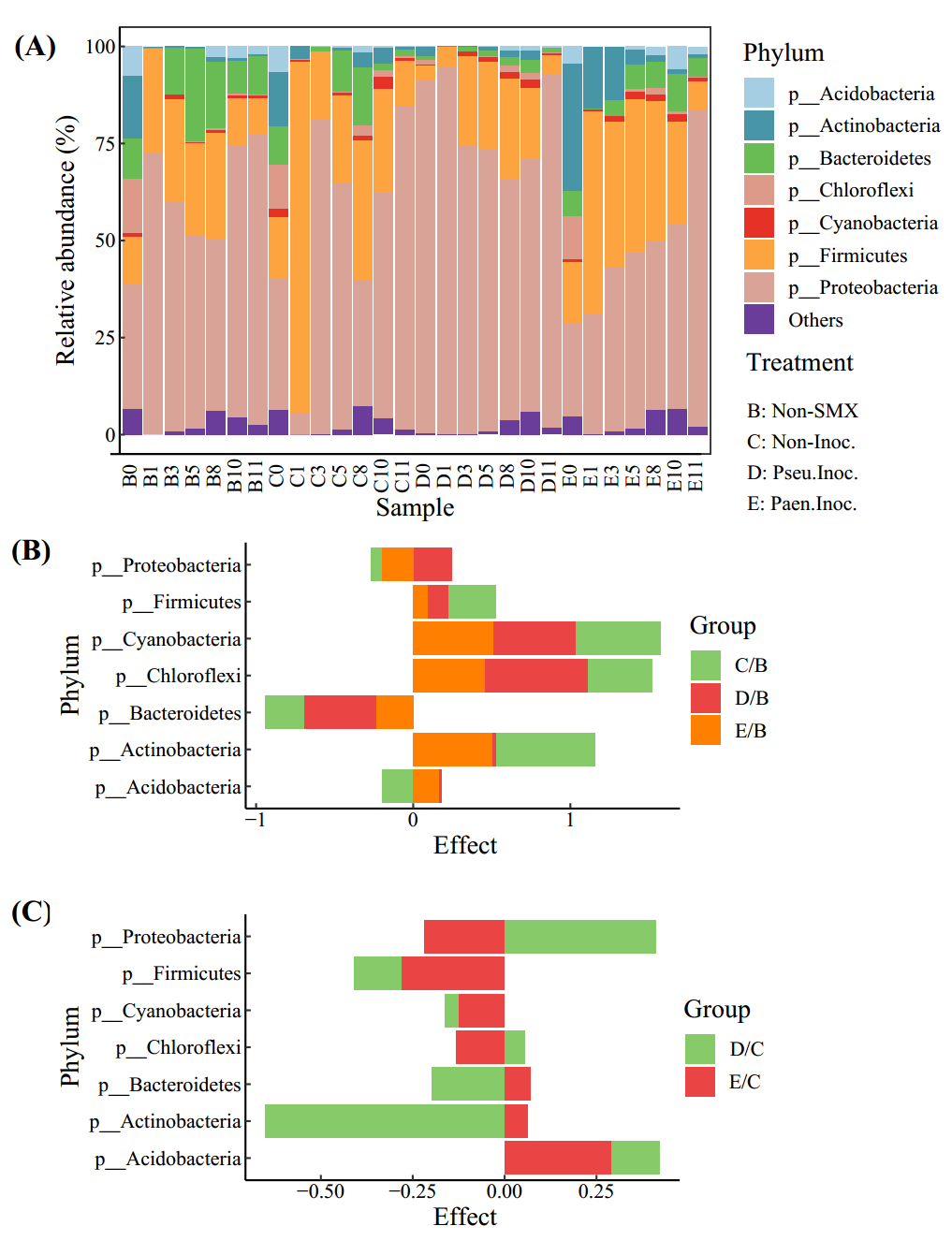


**Fig. S1.** Relative abundance of the dominant phyla (all taxonomic groups except for the top 7 were merged into the “Others” group) **(A).** Comparison of the phylum distribution between SMX-amended treatments (treatments C, D and E) and non-SMX-amended control (treatment B) **(B)** and between inoculation treatments (treatments D and E) and non-inoculation treatment (treatment C) **(C).**


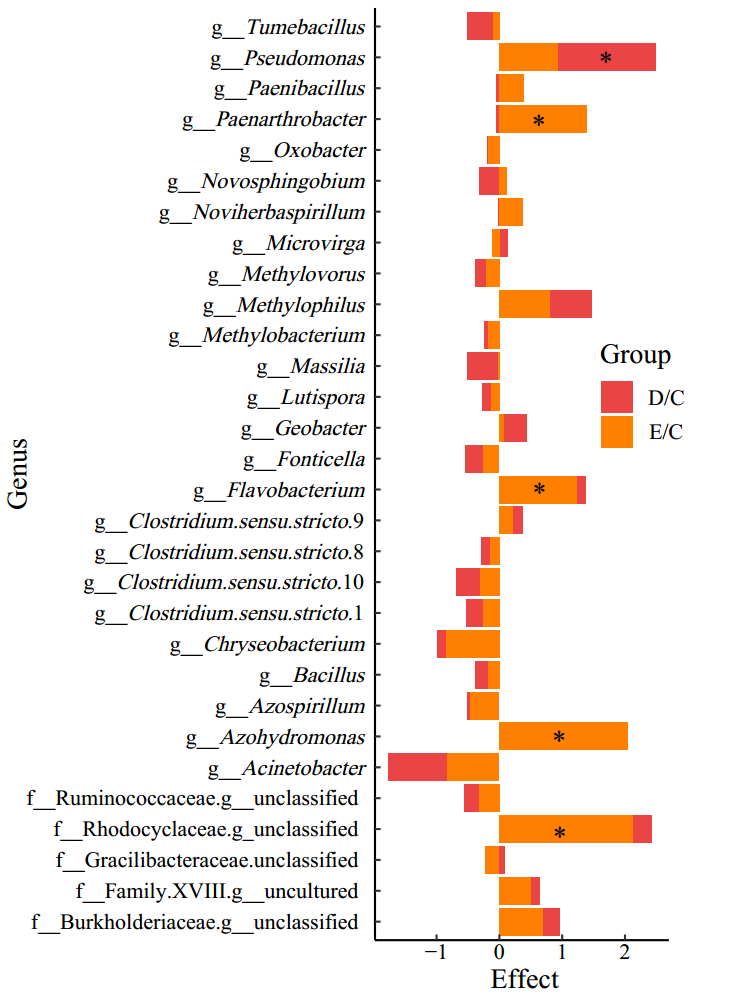


**Fig. S2.** Comparison of the different genus distribution between inoculation treatments (treatments D and E) and non-inoculation treatment (treatment C).


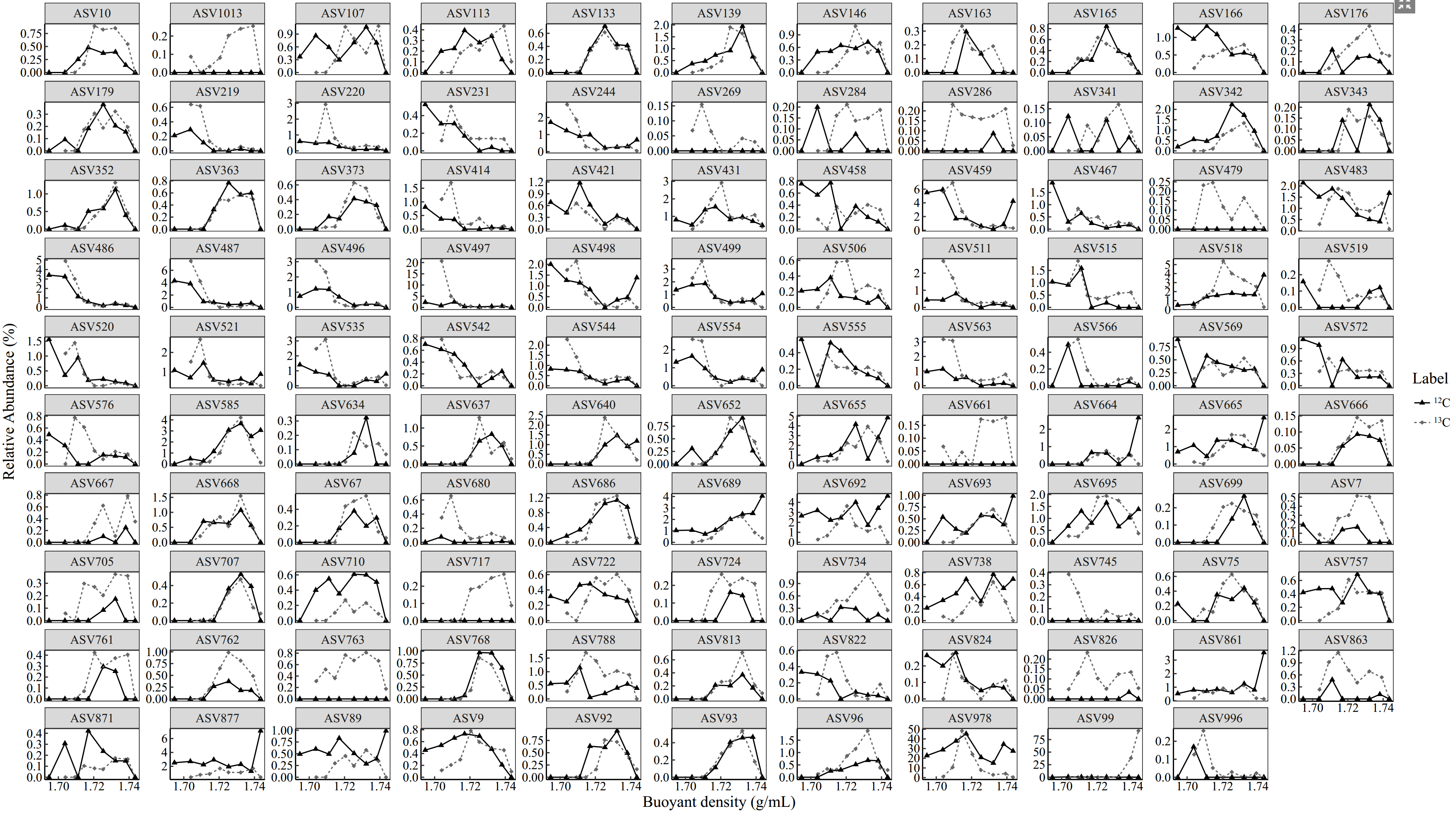


**Fig. S3.** Relative abundance of ASVs along buoyant density gradients from the treatment inoculated with *Pseudomonas* sp. M2.


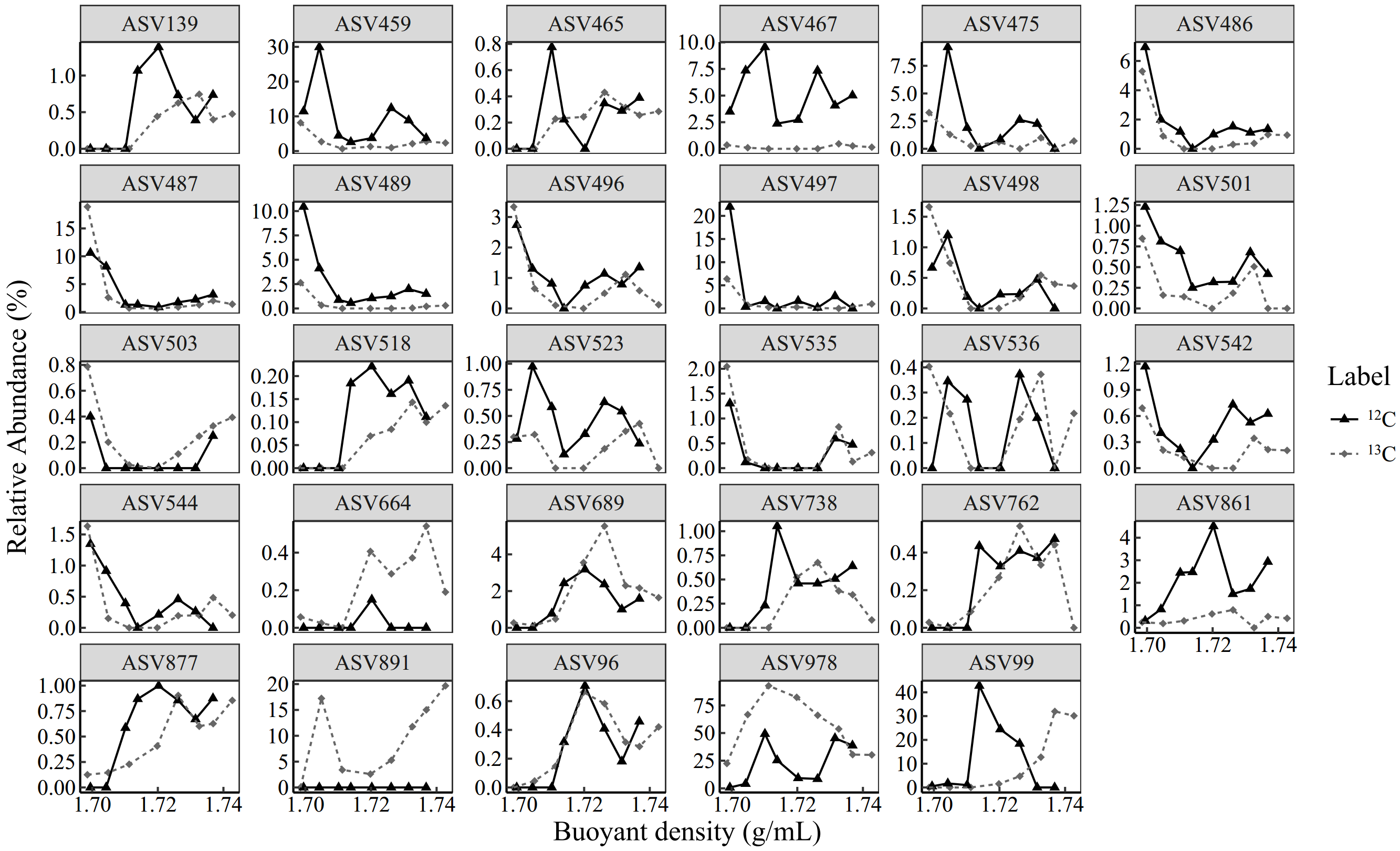


**Fig. S4.** Relative abundance of ASVs along buoyant density gradients from the treatment inoculated with *Paenarthrobacter* sp. R1.


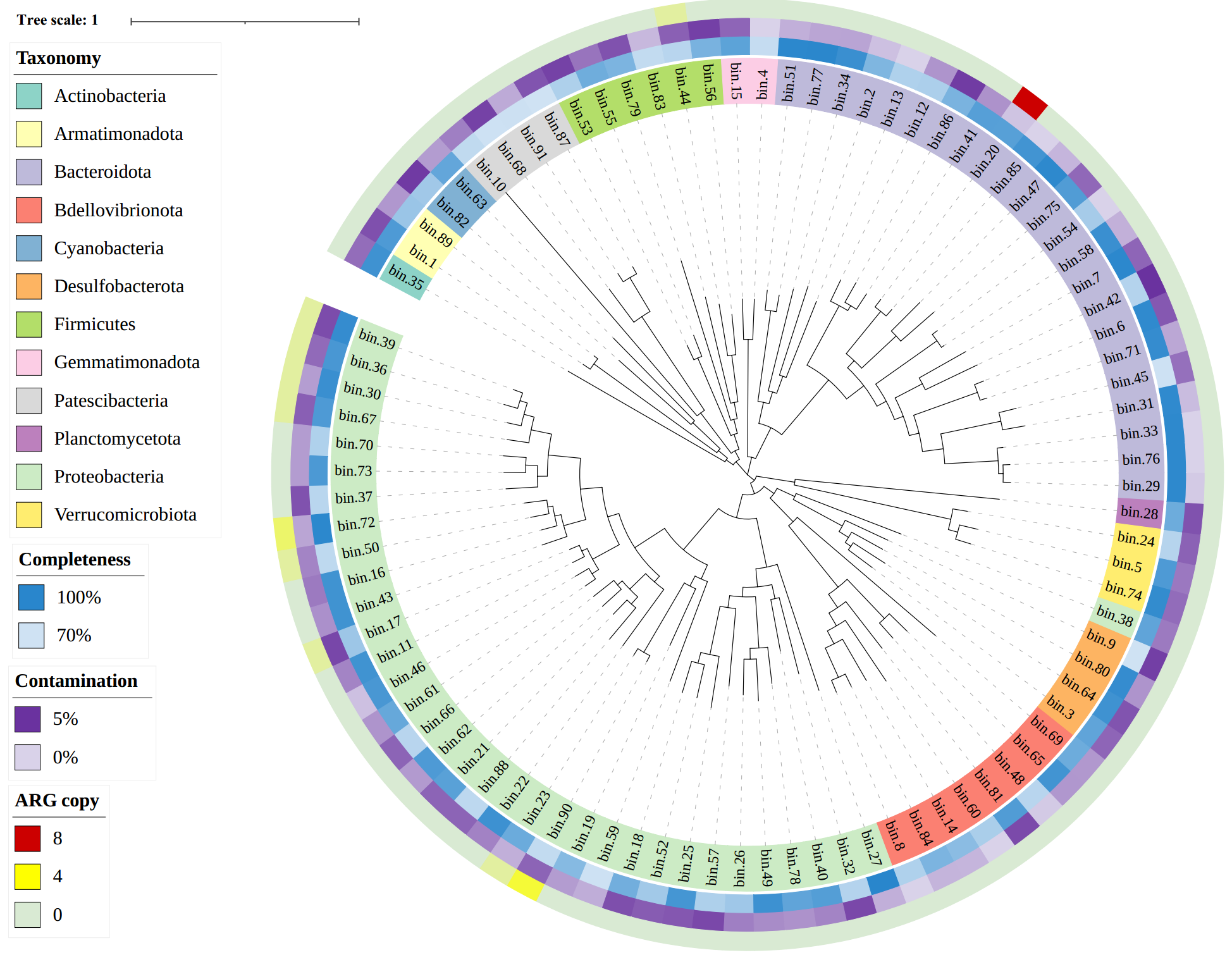


**Fig. S5**. Phylogenetic tree using single-copy gene of all assembled bins.
